# *NOS3* Gene Polymorphisms (rs2070744 and rs1799983) and Differentiated Thyroid Cancer: Investigating Associations with Clinical Outcomes

**DOI:** 10.3390/ijms26020759

**Published:** 2025-01-17

**Authors:** Robert Aurelian Tiucă, Raluca Monica Pop, Oana Mirela Tiucă, Claudia Bănescu, Ana Claudia Cârstea, Cristina Preda, Ionela Maria Pașcanu

**Affiliations:** 1 Doctoral School of Medicine and Pharmacy, George Emil Palade University of Medicine, Pharmacy, Science, and Technology of Targu Mures, 540142 Targu Mures, Romania; 2 Department of Endocrinology, George Emil Palade University of Medicine, Pharmacy, Science, and Technology of Targu Mures, 540142 Targu Mures, Romania; 3 Compartment of Endocrinology, Mures County Clinical Hospital, 540139 Targu Mures, Romania; 4 Department of Dermatology, George Emil Palade University of Medicine, Pharmacy, Science, and Technology of Targu Mures, 540142 Targu Mures, Romania; 5 Dermatology Clinic, Mures County Clinical Hospital, 540015 Targu Mures, Romania; 6 Department of Medical Genetics, George Emil Palade University of Medicine, Pharmacy, Science, and Technology of Targu Mures, 540142 Targu Mures, Romania; 7 Center for Advanced Medical and Pharmaceutical Research, Genetics Laboratory, George Emil Palade University of Medicine, Pharmacy, Science, and Technology of Targu Mures, 540142 Targu Mures, Romania; 8 Medical Genetics Laboratory, Emergency County Hospital of Targu Mures, 540136 Targu Mures, Romania; 9 Department of Endocrinology, University of Medicine and Pharmacy “Grigore T. Popa”, 700115 Iasi, Romania; 10 Department of Endocrinology, ‘Sf. Spiridon’ County Hospital, 700111 Iasi, Romania

**Keywords:** *NOS3* polymorphisms, differentiated thyroid cancer, genetic biomarkers, single-nucleotide polymorphisms, cancer genetics

## Abstract

Differentiated thyroid cancer (DTC) is the most common endocrine malignancy, with genetic factors playing an important role in its development and progression. This study investigated the association between nitric oxide synthase 3 (*NOS3*) gene polymorphisms (−786T>C or rs2070744 and Glu298Asp or c.894T>G or rs1799983) and the clinical characteristics and outcomes of DTC, aiming to evaluate their potential as biomarkers for prognosis. A case-control study was conducted, enrolling 172 individuals from the Endocrinology Clinics of Târgu Mureș and Iași, Romania, between 2021 and 2023. This study included 88 patients with DTC and 84 healthy controls, matched for age and sex. DNA was extracted from blood samples, and the *NOS3* polymorphisms were genotyped using TaqMan assays. Statistical analysis included chi-square tests with a significance level set at *p* < 0.05. The distribution of the rs2070744 and rs1799983 polymorphisms showed no significant differences between the patients with DTC and healthy controls (*p* = 0.387 and *p* = 0.329, respectively). Furthermore, no significant associations were found between these polymorphisms and key clinical outcomes such as biochemical control, structural control, or loco-regional metastases. Our findings indicate that *NOS3* rs2070744 and rs1799983 gene polymorphisms do not significantly influence the clinical outcomes of DTC, suggesting their limited utility as biomarkers for DTC prognosis.

## 1. Introduction

Thyroid cancer is the most common endocrine malignancy worldwide, mainly originating from follicular cells [1]. Follicular-cell-derived thyroid carcinomas include papillary thyroid carcinoma (PTC), follicular thyroid carcinoma (FTC), oncocytic carcinoma (OCA), differentiated high-grade thyroid carcinoma (DHGTC), invasive encapsulated follicular variant PTC (IEFV-PTC), poorly differentiated thyroid carcinoma (PDTC), and anaplastic thyroid carcinoma (ACA) [2]. PTC and FTC are the most frequent histological types commonly referred to as differentiated thyroid cancer (DTC). PTC is the most prevalent histological type, making up over 80% of cases, and has the best prognosis, though some aggressive subtypes may have poorer outcomes [3,4]. DTC is the most common in middle-aged women, with recent studies suggesting a women-to-men incidence ratio of 4.39:1 for subclinical PTC smaller than 2 cm [5]. Notably, autopsy and epidemiological studies found a rate close to 1:1 for all other sizes and types of thyroid cancer [5]. This phenomenon could be explained by the higher detection rate of thyroid nodules among women compared to men, which leads to more frequent diagnostic work-ups and, thus, to higher rates of subclinical thyroid cancer [6]. Although most cases of DTC have a good prognosis, it may invade the lymphatic system, leading to multifocality, lymph node, and distant metastases [3,4].

Over the past decades, research has investigated genetic mutations linked to thyroid cancer, with early studies revealing only a small fraction of genetic modifications [7,8]. However, thanks to advancements in next-generation sequencing, the understanding of thyroid cancer’s genomic landscape has dramatically increased [9]. In DTC, the key genes responsible for tumorigenesis are involved in two main pathways: the MAPK pathway, which influences gene expression, proliferation, differentiation, and apoptosis, and the PI3K-AKT pathway, which regulates glucose metabolism as well as cell survival, adhesion, and mobility [9,10,11]. MAPK pathway mutations, such as BRAF and RAS, increase the likelihood of PTC occurrence, evolution, and prognosis, while the PI3K-AKT pathway leads to FTC through mutations like RAS, PIK3CA, and AKT1. TERT and p53 mutations in these histological types have been linked to greater aggressiveness and increased risk of progression to PDTC and/or ACA [9,10,11].

In addition to the two signaling pathways commonly implicated in thyroid cancer, recent research has identified several epigenetic modifications associated with thyroid carcinogenesis. These include histone modifications that affect chromatin’s structure, the aberrant DNA methylation of tumor suppressor genes, and the epigenetic modulation of non-coding RNAs, such as microRNAs (miRNAs) [12]. For instance, hypermethylation of the promoter region of the tumor suppressor gene Ras association domain family 1 A (RASSF1A) has been linked to DTC progression and more aggressive forms [13,14]. Furthermore, aberrant miRNA profiles, such as the overexpression of miR-146b and miR-221, have been associated with aggressive tumor behavior and poor prognosis [12,14,15,16]. These epigenetic modifications add complexity to the molecular landscape of thyroid cancer, and insights regarding these biological mechanisms may provide new targetable molecular markers for clinical use in both the diagnosis and prognosis of DTC.

Recently, various single-nucleotide polymorphisms (SNPs) linked to the development of DTC have been studied [17]. For instance, a meta-analysis identified 19 SNPs as significantly associated with thyroid cancer susceptibility, out of which strong associations were identified for 7 SNPs: *POU5F1B* rs6983267, *FOXE1* rs966423, *TERT* rs2736100, *NKX2-1* rs944289, *FOXE1* rs1867277, *FOXE1* rs2439302, and *RET* rs1799939 [18]. Additionally, other SNPs in genes such as *GLP1R* (rs1042044 and rs6923761), *BTG3* (rs9977638), *CASP9* (rs884363), *LRP4* (rs898604), and *PCNXL2* (rs10910660) have been associated with tumor aggressiveness, metastasis, and PTC susceptibility [19]. SNPs are variations in the DNA sequence commonly found in the general population. The primary mechanisms contributing to the occurrence of SNPs include point mutations and chromosomal rearrangements. Other mechanisms, such as nucleotide substitutions, deletions, and insertions, have also been described [19].

The *NOS3* gene encodes nitric oxide synthase 3 (NOS3), an enzyme primarily responsible for the production of nitric oxide (NO) from L-arginine within endothelial cells [20]. NO has numerous physiological roles, being involved in neuronal signaling, vasodilation, or blood pressure regulation [20]. Beyond these roles, NO inhibits cell proliferation, prevents leukocyte adhesion, reduces platelet aggregation, and promotes angiogenesis [20]. It also exerts anti-inflammatory and antioxidant effects by stimulating the expression of superoxide dismutase, an enzyme that neutralizes harmful superoxide radicals [20]. Although NOS3 produces NO in smaller quantities, its effects significantly maintain vascular health and overall physiological homeostasis [20]. Research indicates that SNPs in the *NOS3* gene are linked to several health issues, including hypertension, migraines, erectile dysfunction, pre-eclampsia, and diabetes complications [21,22,23,24,25]. While NO usually has anti-inflammatory effects, these genetic variations might disrupt its physiological function and production, potentially making NO pro-inflammatory and reducing its antioxidant action, which can damage DNA, RNA, and proteins, thus increasing cancer risk [26].

**Figure 1:**
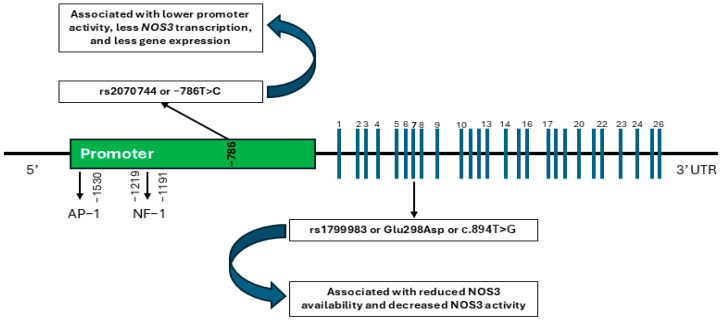
Schematic representation of *NOS3* gene. This gene contains a promoter region, 25 introns, and 26 exons (represented by blue lines). The promoter region contains transcription factor binding sites such as AP-1 and NF-1. The functional polymorphisms rs2070744 in the promoter region and rs1799983 in exon 7 are illustrated in this figure.

The SNP rs1799983, located in exon 7 of the *NOS3* gene, leads to a Glu298Asp substitution in the protein, reducing NOS3 binding to caveolin-1 and lowering its availability in endothelial cells (Figure 1). This may result in reduced NOS3 activity and NO production [20]. Although the two alleles of the rs1799983 polymorphism exhibit comparable enzymatic activity, the T allele has been reported to undergo selective proteolysis, potentially lowering NOS3 levels [27,28]. Moreover, some studies proposed that the specific cleavage of the T allele may be an artifact, suggesting that the functional effects of the rs1799983 variation might instead arise from disrupted caveolar localization of NOS3 [29]. Since glutamic acid and aspartic acid are conservative substitutions, it has also been proposed that this polymorphism may act as a marker for a functional effect elsewhere in the gene or its surrounding region rather than exerting a direct impact itself [29,30]. SNP rs2070744 is located in the promoter region of the *NOS3* gene and affects its transcriptional activity (Figure 1). The cytosine replacement at position −786 reduces *NOS3* transcription by increasing the binding affinity of the repressor protein RPA1 to the promoter. In vivo studies show lower circulating NO-related markers in individuals with the C allele, supporting the functional impact of this SNP [20,31]. Notably, these two *NOS3* gene SNPs have been studied in relation to cancers such as breast, prostate, colorectal, and lung cancers, with evidence suggesting that these SNPs may influence tumor progression by modulating NO production and vascularity [32,33,34,35]. Based on these findings, it is worth investigating whether these SNPs similarly contribute to thyroid cancer development and/or outcomes.

The relationship between *NOS3* gene polymorphisms and thyroid cancer has not been clearly established, with only a limited number of studies conducted in this area. To date, the *NOS3* intron 4 polymorphism has been the only variant studied in relation to PTC [36]. Our study’s purpose was to examine, for the first time, the impact of *NOS3* −786T>C or rs2070744 and Glu298Asp or c.894T>G or rs1799983 gene polymorphisms (Figure 1) on the clinical characteristics and outcomes of DTC. By analyzing these genetic variations, we aimed to uncover their potential implications for DTC’s diagnosis and prognosis.

## 2. Results

This study included 172 patients divided into a case group of 88 subjects diagnosed with DTC and a control group of 84 healthy individuals. Most subjects included in the case group were women (85.2%), with a mean age at the last evaluation of 55.46 ± 13.65 years old. The median follow-up time from DTC diagnosis was 5 years (4.0–7.6). Most patients were non-smokers (71.6%), underwent total thyroidectomy (98.9%), had an indolent histological type of DTC (86.4%), and had a history of RAI therapy (77.3%). Regarding the post-therapeutic evolution, the majority of patients had good biochemical (70.5%) and structural (73.9%) control, with few cases developing metastases (6.8%). The clinical characteristics of the study population are illustrated in Table 1.

The distributions of the genotypes in the population were in Hardy–Weinberg equilibrium (rs2070744: *p* = 0.589; rs1799983: *p* = 0.219). Genotype information was available for 100% of the cases (88/88) and 100% of the controls (84/84). No significant differences regarding the genotypes were found between the two studied groups. The distribution of genotype frequencies in patients with DTC and the population controls is illustrated in Table 2.

The association between the *NOS3* gene rs1799983 and rs2070744 polymorphisms and the risk of DTC was assessed (Table 3). Neither the rs1799983 nor the rs2070744 polymorphism of the *NOS3* gene was significantly related to DTC susceptibility.

We found no statistically significant relationship between sex and rs1799983 genotypes or rs2070744 genotypes. The distribution of genotype frequencies in the whole study population according to sex did not differ significantly (Table 4).

No statistically significant associations were found between the rs1799983 genotype and male sex (*p* = 0.982), smoking (*p* = 0.354), aggressive histological type (*p* = 0.944), or incomplete biochemical (*p* = 0.592) and structural (*p* = 0.204) control. Furthermore, no statistically significant associations were found between rs1799983 genotype and history of RAI therapy (*p* = 0.856) and occurrence of loco-regional and/or distance metastases (*p* = 0.725) (Table 5).

Regarding the rs2070744 genotype, similar results were found, with no statistically significant associations between this polymorphism’s genotypes and male sex (*p* = 0.566), smoking (*p* = 0.958), aggressive histological type (*p* = 0.349), incomplete biochemical (*p* = 0.907) and structural (*p* = 0.382), history of RAI therapy (*p* = 0.436), and occurrence of loco-regional and/or distance metastases (*p* = 0.246) (Table 5).

Furthermore, no significant difference was found between rs1799983 homozygous wild-type (TT) and homozygous variant (GG) genotypes regarding male sex, smoking, aggressive histological type, history of RAI, incomplete biochemical and structural control, and occurrence of loco-regional and/or distance metastases (Table 6). Similar results were found for the rs2070744 homozygous wild-type (CC) and homozygous variant (TT) genotypes when analyzing the same clinicopathological characteristics (Table 6).

Table 7 outlines the analysis of the predictive factors for incomplete biochemical control. Among the examined variables, incomplete structural control was strongly associated with incomplete biochemical control, with an OR of 8.707 (95% CI: 2.154–35.193; *p* = 0.002). Male sex also emerged as a significant predictive factor, with an OR of 10.240 (95% CI: 1.908–54.959; *p* = 0.007). Additionally, a history of RAI therapy was found to be protective, with an OR of 0.130 (95% CI: 0.028–0.595; *p* = 0.009).

## 3. Discussion

This study investigated whether the *NOS3* gene polymorphisms rs2070744 and rs1799983 influence the clinical characteristics and evolution of patients diagnosed with DTC. Our findings indicate no significant association between these *NOS3* polymorphisms and either the susceptibility to DTC (Table 3) or the progression and clinical outcomes of the disease (Table 7), despite the established roles of NOS3 in NO production and the increased cancer susceptibility that *NOS3* polymorphisms might promote. This might be explained by the complex oncogenesis of thyroid cancer, where molecular pathways, such as MAPK and PI3K-AKT, are the primary mechanisms involved in tumorigenesis [7,8,9]. To the best of our knowledge, this study is the first to evaluate these *NOS3* gene polymorphisms in relation to DTC, making this work a pioneer in its field.

Cerqueira et al. identified a significant association between the *NOS3* intron 4 polymorphism and susceptibility to PTC [36]. Cerqueira et al. found a significant difference in the genotypic distribution between patients with PTC and healthy individuals, identifying the bb genotype as a protective factor against PTC (*p* < 0.001, OR: 0.16, CI 95%: 0.06–0.42). Furthermore, the presence of the a allele was associated with a significantly increased risk of developing PTC (*p* < 0.001, OR: 3.54, 95%CI: 1.86–6.73). However, the study found no associations between *NOS3* intron 4 polymorphisms and several clinical characteristics [36]. The previously mentioned study was the first to investigate the association between *NOS3* intron 4 polymorphisms and PTC susceptibility, creating a basis for future studies to investigate the relationship between *NOS3* gene polymorphisms and thyroid cancer. Interestingly, our study did not observe any protective or risk-related effects of the *NOS3* rs2070744 and rs1799983 gene polymorphisms in patients with DTC. This suggests a potential difference in how *NOS3* polymorphisms affect different thyroid cancer subtypes, considering that we investigated these polymorphisms in more than just the papillary subtype of thyroid cancer. For example, PTC and FTC show distinct patterns of angiogenesis, oxidative stress, and inflammatory responses, which are processes regulated by NO [37,38,39,40,41]. The functional effects of *NOS3* polymorphisms—such as altered NO production—could influence these pathways differently in each subtype, potentially contributing to subtype-specific cancer behaviors.

The *NOS3* gene has been linked to various cancers and cardiovascular diseases due to its role in NO synthesis and the regulation of vascular tone. However, the exact contribution of specific *NOS3* polymorphisms to cancer susceptibility is still under investigation. *NOS3* rs2070744 and rs1799983 gene polymorphisms have been investigated in relationship with other types of cancers, with conflicting results. The main reasons for the ongoing debate about the impact of *NOS3* gene polymorphisms on cancer are mainly due to several factors, such as the number of patients included in such research, cancer type, genetic background, and the influence of ethnic variability and environmental factors [42].

The logistic regression analysis for therapeutic outcomes, specifically incomplete biochemical control, identified the male sex as a significant predictor (*p* = 0.007; OR = 10.240), while a history of RAI therapy was found to be a significant protective factor (*p* = 0.009; OR = 0.130). In contrast, the studied SNPs did not demonstrate statistical significance (Table 7). Male sex has previously been associated with adverse clinicopathological characteristics in DTC, including lymph node metastases and increased tumor size [43]. These factors may contribute to the higher likelihood of men with DTC experiencing incomplete biochemical control, which could lead to an elevated risk of tumor recurrence or metastases. The protective effect of RAI after thyroidectomy in DTC is likely due to its ability to ablate residual thyroid tissue and eliminate microscopic disease, leading to lower thyroglobulin levels [44]. Interestingly, the absence of significant associations between *NOS3* SNPs and therapeutic response in this study contrasts the findings in other cancers, where *NOS3* polymorphisms have been shown to influence treatment outcomes or prognosis.

Ryk et al. conducted a study investigating whether these SNPs confer protection or susceptibility to urinary bladder cancer [45]. The study identified that the *NOS3* promoter polymorphism −786T>C (rs2070744) may influence bladder cancer risk, while Glu298Asp (rs1799983) carries no increased bladder cancer risk [45]. According to a meta-analysis and case–control study conducted by Gao et al., rs2070744 and rs1799983 polymorphisms might be associated with an increased risk of breast cancer, particularly in specific populations and age groups [46]. Brankovic et al.’s study results suggest that *NOS3* gene polymorphisms (−786T>C, −764A>G, −714G>T, −690C>T, −649G>A, and 894G>T) confer genetic susceptibility for the progression of prostate cancer [47]. Zhu et al.’s meta-analysis pointed out that rs2070744 polymorphisms might represent a susceptible factor for gastric cancer [48]. These studies indicate that *NOS3* gene polymorphisms have a diverse impact on cancer risk and progression, often depending on the type of cancer, the specific polymorphism involved, ethnicity, and environmental exposure of the population included in the analysis. In contrast to these findings, Haque et al. found no significant association between rs1799983 polymorphisms and increased or decreased risk of overall cancer [49]. As mentioned earlier, numerous tumor-related and patient-related factors impact the effects of these polymorphisms. Therefore, future research should focus on prospective studies with larger cohorts to have more relevant conclusions that could be applied to a more significant population.

The lack of association between *NOS3* gene polymorphisms and DTC in our study might be explained by the complex oncogenesis of DTC. Genetic factors, environmental influences, and molecular pathways influence DTC risk and progression [50]. Moreover, other factors that may explain our results are the difference in DTC subtypes, the ethnicity of our subjects, or the sample size. Given the results of our study, *NOS3* rs2070744 and rs1799983 gene polymorphisms are unlikely to serve as reliable biomarkers for DTC susceptibility or for predicting the clinical course of the disease. Nevertheless, it remains possible that NOS3-mediated NO production influences thyroid cancer oncogenesis via different pathways that were not explained by the polymorphisms examined in our study. Furthermore, it is possible that epigenetic modifications of the *NOS3* gene could impact thyroid cancer risk, considering the recent studies that showed a complex relation between epigenetic modifications and thyroid cancer [13,14,51]. Future research should investigate *NOS3* gene expression and epigenetic regulation in patients with DTC to uncover potential mechanisms of NO involvement in thyroid tumorigenesis. In the absence of experimental studies directly investigating the role of NOS3 in thyroid cancer, future research could involve murine models and CRISPR-Cas9 gene editing technologies to explore the functional implications of *NOS3* gene polymorphisms. For instance, NOS3 knockout mice could help determine the impact of NO depletion on tumor angiogenesis and progression. At the same time, CRISPR-edited cancer cell lines could assess how specific polymorphisms, such as rs1799983 or rs2070744, affect NO production, oxidative stress, and inflammatory responses. Such studies would provide mechanistic insights into the role of NOS3 in cancer biology and complement existing association-based findings.

Our study had several limitations. First, the relatively small sample size may have limited the statistical power to detect subtle genetic effects of the *NOS3* polymorphisms. Moreover, our study did not include functional assays to confirm the impact of rs1799983 and rs2070744 on NOS3 enzymatic activity or NO production. Future research could use large publicly available genomic datasets, such as The Cancer Genome Atlas (TCGA), to validate and expand our findings. The TCGA-THCA dataset, which includes whole-genome and whole-exome sequencing data from a large cohort of patients with thyroid cancer, mainly PTC, offers an opportunity to assess the prevalence and clinical relevance of *NOS3* polymorphisms, such as rs1799983 and rs2070744. Resources such as the International Cancer Genome Consortium (ICGC) and the Genotype-Tissue Expression (GTEx) project could help explore the associations between *NOS3* polymorphisms, gene expression, and thyroid cancer phenotypes. These approaches would provide greater statistical power and broader insights into the role of NOS3 in thyroid cancer biology. Second, our study focused on two common *NOS3* polymorphisms, potentially overlooking other genetic variants or epigenetic factors that may influence DTC susceptibility and progression. Future research could include more detailed analyses of additional *NOS3* variants and investigate the role of epigenetic modifications, such as DNA methylation, histone modifications, or epigenetic modulation of non-coding RNAs, in regulating *NOS3* expression and activity. Third, the lack of ethnically diverse populations in our study limits the generalizability of our findings. Our findings may not be generalizable to other populations or healthcare settings as a double-center study. Multi-center studies are required to validate these results across diverse cohorts. More extensive, multi-center studies with more ethnically diverse cohorts are needed to understand better the genetic and environmental interactions influencing DTC development. Furthermore, as our study was cross-sectional, it is limited in establishing causal relationships between these *NOS3* polymorphisms and thyroid cancer outcomes. Assessing structural control in a retrospective analysis is challenging due to potential biases in interpreting prior thyroid ultrasound results, which are influenced by clinician expertise and may be inconsistent across evaluations. Benign conditions mimicking incomplete structural control, such as scars or reactive lymphoid hyperplasia, further complicate accurate classification. Finally, our study did not explore the potential interactions between *NOS3* polymorphisms and other molecular markers involved in thyroid cancer pathogenesis. Future studies should consider these interactions to provide a broader view of the genetic landscape contributing to DTC, thus increasing the likelihood of more conclusive results.

## 4. Materials and Methods

### 4.1. Study Design

This case–control study enrolled patients with DTC who were admitted to the Endocrinology Clinic of Târgu Mureș, Romania, and the Endocrinology Clinic of Iași, Romania, between 2021 and 2023. This study included 172 individuals. Subjects were divided into two groups:

(a) Case group with documented DTC diagnoses ranging from 1978 to 2022.
  I. Inclusion criteria: patients over 18 years old with a confirmed pathological diagnosis of DTC.
  II. Exclusion criteria: incomplete information on the pathological examination, as well as missing information about the biochemical and structural responses following therapy.
(b) Control group
  I. Inclusion criteria: subjects over 18 years old.
  II. Exclusion criteria: personal history of any thyroid disease, personal history of any malignant disease.

### 4.2. Data Collection

The data were gathered from the hospital’s electronic databases and patients’ medical records. For each patient, information was extracted on sex, age, behaviors such as smoking, type of surgical treatment, tumor’s histological type, history of radioactive iodine (RAI) treatment, biochemical and structural responses following treatment, tumor recurrence, and the presence or occurrence of loco-regional or distant metastases before and after treatment (Table 8).

The laboratory parameters analyzed to assess the biochemical response to therapy included the latest measurements of thyroid-stimulating hormone (TSH), thyroglobulin, and anti-thyroglobulin antibodies (TgAb). Thyroid ultrasound results were used to evaluate the structural response to therapy.

The histological types were classified into two categories:

(a) Indolent histological types: classic papillary carcinoma, papillary microcarcinoma, follicular variant of papillary microcarcinoma, follicular carcinoma, and Warthin-like variant.
(b) Aggressive histological types: tall-cell variant, poorly differentiated component of follicular carcinoma, follicular carcinoma with insular carcinoma component, oncocytic carcinoma, and Hobnail variant.

### 4.3. Response to Therapy

Biochemical incomplete response at the last evaluation was defined as a stimulated thyroglobulin (stTg) value greater than 1 ng/mL or a non-stimulated thyroglobulin (nstTg) value greater than 0.2 ng/mL in patients who had undergone total thyroidectomy and RAI ablation; an stTg value greater than 2 ng/mL or nstTg value greater than 0.2 ng/mL in patients who had only undergone total thyroidectomy; and an nstTg value greater than 30 ng/mL in patients who had undergone isthmolobectomy. An incomplete structural response was defined as the ultrasound presence of hypoechoic or isoechoic residual tissue in the thyroid bed, the presence of suspicious laterocervical lymph nodes, or distant metastasis [52,53].

### 4.4. Laboratory Methods

After providing written informed consent, each participant had 4 mL of venous blood drawn into EDTA tubes, which was subsequently frozen at −20 °C until DNA could be extracted. DNA was extracted from these samples using a PureLink Genomic DNA Kit (Thermo Fisher Scientific, Waltham, MA, USA). The genetic variants analyzed included rs1799983, a missense mutation located in exon 7 of the *NOS3* gene, and rs2070744, found in the promoter region of *NOS3* (Figure 1) [20]. DNA was analyzed using TaqMan assays on a real-time PCR system to determine the genotype of these variants. According to previous reports the following should be noted for these polymorphisms [54,55]:

- rs1799983: T = wild-type allele; G = variant allele; TT = homozygous wild-type; GG = homozygous variant; GT = heterozygous variant.
- rs2070744: C = wild-type allele; T = variant allele; CC = homozygous wild-type; TT = homozygous variant; CT = heterozygous variant.

### 4.5. Statistical Analysis

Microsoft Excel (Microsoft Corporation, Redmond, WA, USA) was used for data collection. Statistical analysis was conducted using IBM SPSS Statistics for Windows, Version 25.0 (IBM Corp., Armonk, NY, USA) and GraphPad Prism v.8.2 (GraphPad Software, Inc., San Diego, CA, USA). The following statistical tests were employed: Kolmogorov–Smirnov normality test, Mann–Whitney test for comparing central tendencies, and chi-square and its variants for testing associations between categorical variables. Fisher’s exact test was used for testing the Hardy–Weinberg equilibrium. Values of two-sided *p* < 0.05 were considered statistically significant.

### 4.6. Ethical Approval

This study was conducted in accordance with the Declaration of Helsinki and was approved by the Ethics Committee of the University of Medicine, Pharmacy, Science, and Technology “George Emil Palade” of Târgu Mureș, under approval number 1506/25 November 2021.

## 5. Conclusions

This study found no significant association between the *NOS3* rs1799983 and rs2070744 gene polymorphisms and the clinical characteristics or outcomes of patients with DTC. Neither polymorphism influenced biochemical control, structural control, or susceptibility to loco-regional or distant metastases. These findings suggest that *NOS3* rs1799983 and rs2070744 gene polymorphisms might not be valuable biomarkers for DTC prognosis or progression. Further research should investigate the interaction of *NOS3* gene polymorphisms with other molecular markers involved in the oncogenesis of thyroid cancer.

## Figures and Tables

**Table 1:** Clinical characteristics of the study population.

**CASE GROUP**	
Number of subjects	88
Sex distribution	F: 75 (85.2%) M: 13 (14.8%)
Mean age at the last evaluation (years)	55.46 ± 13.65
Median follow-up time (years)	5 (4.0–7.6)
Smoking status	NS: 63 (71.6%) S: 25 (28.4%)
Type of surgical intervention	TT: 87 (98.9%) IL: 1 (1.1%)
Histological type of DTC	Indolent: 76 (86.4%) Aggressive: 12 (13.6%)
History of RAI administration	Yes: 68 (77.3%) No: 20 (22.7%)
Loco-regional or distant metastases at diagnosis	Yes: 26 (29.5%) No: 62 (70.5%)
Good biochemical control at last evaluation	Yes: 62 (70.5%) No: 26 (29.5%)
Good structural control at last evaluation	Yes: 65 (73.9%) No: 23 (26.1%)
Loco-regional or distant metastases after therapy	Yes: 6 (6.8%) No: 82 (93.2%)
**CONTROL GROUP**	
Number of subjects	84
Sex distribution	F: 68 (81%) M: 16 (19%)
Mean age	54.41 ± 13.59

Abbreviations: DTC, differentiated thyroid cancer; F, female; M, male; RAI, radioactive iodine; NS, non-smoker; S, smoker; TT, total thyroidectomy; IL, isthmolobectomy.

**Table 2:** Distribution of the genotype of *NOS3* rs2070744 and rs1799983 polymorphisms in patients with DTC and control subjects.

Polymorphism	Genotype	DTC Cases *n* (%)	Population Controls *n* (%)	*p* Value
rs2070744	CC (homozygous wild type)	16 (18.2%)	13 (15.5%)	0.387
	CT (heterozygous variant)	39 (44.3%)	46 (54.7%)	
	TT (homozygous variant)	33 (37.6%)	25 (29.8%)	
	C (allele)	71 (40.3%)	72 (42.9%)	0.716
	T (allele)	105 (59.7%)	96 (57.1%)	
rs1799983	TT (homozygous wild type)	8 (9.0%)	8 (9.5%)	0.329
	GT (heterozygous variant)	40 (45.5%)	29 (34.5%)	
	GG (homozygous variant)	40 (45.5%)	47 (56.0%)	
	G (allele)	120 (68.2%)	123 (62.4%)	0.292
	T (allele)	56 (31.8%)	74 (37.6%)	

Abbreviations: DTC, differentiated thyroid cancer; *n*, number.

**Table 3:** *NOS3* rs1799983 and rs2070744 polymorphisms and DTC susceptibility.

Variable	OR	95% CI	*p* Value
rs1799983 wild-type TT genotype			0.191
rs1799983 heterozygous variant GT genotype	1.870	0.953–3.668	0.069
rs1799983 homozygous variant GG genotype	1.377	0.461–4.113	0.567
rs2070744 wild-type CC genotype			0.224
rs2070744 heterozygous variant CT genotype	0.701	0.298–1.650	0.416
rs2070744 homozygous variant TT genotype	1.299	0.511–3.301	0.582

Abbreviations: OR, odds ratio; 95% CI, 95% confidence interval.

**Table 4:** Distribution of the genotype of *NOS3* rs2070744 and rs1799983 polymorphisms in patients with DTC and control subjects according to sex.

Polymorphism	Genotype	DTC Cases *n* (%)	*p* Value	Population Controls *n* (%)	*p* Value
rs2070744	CC (homozygous wild type)	F: 13 (17.3%) M: 3 (23.1%)	0.566	F: 11 (16.2%) M: 2 (12.5%)	0.787
	CT (heterozygous variant)	F: 35 (46.7%) M: 4 (30.8%)		F: 36 (52.9%) M: 10 (62.5%)	
	TT (homozygous variant)	F: 27 (36.0%) M: 6 (46.1%)		F: 21 (30.9%) M: 4 (25.0%)	
rs1799983	TT (homozygous wild type)	F: 7 (9.3%) M: 1 (7.7%)	0.982	F: 6 (8.8%) M: 2 (12.5%)	0.080
	GT (heterozygous variant)	F: 34 (45.3%) M: 6 (46.1%)		F: 20 (29.4%) M: 9 (56.2%)	
	GG (homozygous variant)	F: 34 (45.3%) M: 6 (46.1%)		F: 42 (61.8%) M: 5 (31.3%)	

Abbreviations: DTC, differentiated thyroid cancer; *n*, number.

**Table 5:** Associations between *NOS3* rs1799983 and rs2070744 gene polymorphisms and clinicopathological characteristics in patients with DTC.

*NOS3* Gene Polymorphism	Clinicopathological Characteristic	*p* Value
rs1799983	Male sex	0.982
	Smoking	0.354
	Aggressive histological type	0.944
	Incomplete biochemical control	0.592
	Incomplete structural control	0.204
	History of RAI therapy	0.856
	Loco-regional/distance metastases	0.725
rs2070744	Male sex	0.566
	Smoking	0.958
	Aggressive histological type	0.349
	Incomplete biochemical control	0.907
	Incomplete structural control	0.382
	History of RAI therapy	0.436
	Loco-regional/distance metastases	0.246

Abbreviations: NOS3, nitric oxide synthase 3; DTC, differentiated thyroid cancer; RAI, radioactive iodine.

**Table 6:** rs1799983 and rs2070744 homozygous wild-type (TT, respectively CC) and homozygous variant (GG, respectively TT) genotypes and their association with clinicopathological characteristics in patients with DTC.

Genotype	Clinicopathological Characteristic	*p* Value
rs1799983 TT versus non-TT	Male sex	0.849
	Smoking	0.295
	Aggressive histological type	0.922
	History of RAI	0.872
	Incomplete biochemical control	0.768
	Incomplete structural control	0.939
	Loco-regional/distant metastases	0.422
rs1799983 GG versus non-GG	Male sex	0.956
	Smoking	0.517
	Aggressive histological type	0.734
	History of RAI	0.642
	Incomplete biochemical control	0.394
	Incomplete structural control	0.092
	Loco-regional/distant metastases	0.817
rs2070744 CC versus non-CC	Male sex	0.620
	Smoking	0.781
	Aggressive histological type	0.510
	History of RAI	0.280
	Incomplete biochemical control	0.660
	Incomplete structural control	0.170
	Loco-regional/distant metastases	0.232
rs2070744 TT versus non-TT	Male sex	0.485
	Smoking	0.855
	Aggressive histological type	0.336
	History of RAI	0.793
	Incomplete biochemical control	0.904
	Incomplete structural control	0.491
	Loco-regional/distant metastases	0.126

Abbreviations: DTC, differentiated thyroid cancer; RAI, radioactive iodine.

**Table 7:** Predictive factors for incomplete biochemical control.

Variable	OR	95% CI	*p* Value
rs1799983 wild-type TT genotype			0.907
rs1799983 heterozygous variant GT genotype	1.342	0.359–5.018	0.662
rs1799983 homozygous variant GG genotype	1.237	0.172–8.883	0.832
rs2070744 wild-type CC genotype			0.989
rs2070744 heterozygous variant CT genotype	1.050	0.213–5.182	0.952
rs2070744 homozygous variant TT genotype	0.946	0.171–5.236	0.949
Incomplete structural control	8.707	2.154–35.193	0.002
Male sex	10.240	1.908–54.959	0.007
Smoking	3.151	0.899–11.049	0.073
Aggressive histological type	3.487	0.634–19.178	0.151
History of RAI	0.130	0.028–0.595	0.009

Abbreviations: OR, odds ratio; 95% CI, 95% confidence interval; RAI, radioactive iodine.

**Table 8:** Data extracted from hospital’s electronic database and/or patients’ medical records.

Biological Data	Post-Therapeutic Evolution
Sex	Tumoral persistence
Age	Tumoral recurrence
Smoking status	Loco-regional or distant metastases
Histological type of the tumor	
Type of underwent treatment	
Presence of loco-regional or distant metastases at diagnosis	

## Data Availability

All data presented can be made available upon request.
